# Exploring the perception of pre-clinical and clinical educators on clinical reasoning: A qualitative study

**DOI:** 10.1371/journal.pone.0320220

**Published:** 2025-03-21

**Authors:** Siti Norashikin Mohd Tambeh, Farah Dayana Zahedi, Mohamad Nurman Yaman

**Affiliations:** 1 Department of Medical Education, Faculty of Medicine, Universiti Kebangsaan Malaysia, Kuala Lumpur, Malaysia; 2 Faculty of Medicine, Universiti Teknologi MARA, Sungai Buloh Campus, Sungai Buloh, Malaysia; 3 Department of Otorhinolaryngology-Head and Neck Surgery, Faculty of Medicine, Universiti Kebangsaan Malaysia, Kuala Lumpur, Malaysia; Ankara University Faculty of Medicine: Ankara Universitesi Tip Fakultesi, TÜRKIYE

## Abstract

**Introduction:**

Educators have differing perception on the definition and conceptualization of clinical reasoning. Even though clinical reasoning is important in making a sound diagnosis and reducing diagnostic error, educators proved to be a barrier in teaching clinical reasoning due to the lack of awareness of their own reasoning process.

**Objectives:**

This study was conducted to investigate the perception and understanding of pre-clinical and clinical educators on what clinical reasoning entails, their experience, and educational strategies in teaching clinical reasoning.

**Method:**

A semi-structured interview was conducted with fifteen educators encompassing pre-clinical (basic science, laboratory-based) and clinical (surgical-based, medical-based, community-based and emergency medicine) educators. The transcribed interview data was then analysed thematically.

**Results:**

Eight main themes were identified. Knowledge and experience were seen as important components in developing clinical reasoning. It was possible to teach clinical reasoning although there were some difficulties thus the need to have a train-the-trainer programme. Early introduction of clinical reasoning with its incorporation in various teaching and learning method; and the involvement of technological advances were also mentioned by the participants. However, pre-clinical educators did not perceive the importance of these technological advances. Role of educators; cognitive and non-cognitive attributes were also important in developing clinical reasoning.

**Conclusion:**

The perception and understanding of pre-clinical and clinical educators on clinical reasoning did not really differ. They believed that clinical reasoning can be taught, and a train-the-trainer program may be of immeasurable benefit.

## Introduction

Clinical reasoning is one of the core competencies that need to be inculcated in medical students as this skill will be applied throughout their career. The lack of clinical reasoning utilization or inability to apply clinical reasoning has been associated with increased diagnostic error [1] which is one of the causes of preventable harms to patients [2]. Clinical reasoning has been described in literature as a process in which health practitioners observe, collect, and interpret features in patients to come up with a sound diagnosis and appropriate management plan [1,3] and this was the working definition of clinical reasoning for this study. The definition and conceptualization of clinical reasoning differs in clinical reasoning experts from various training backgrounds and professions as they have different ideas on which component was considered relevant to clinical reasoning [4]. Despite its predominant application in clinical years, the development of clinical reasoning begins much earlier during which basic scientific knowledge is obtained, gathered, organized, and stored.

Experts in the medical community agree on the importance of clinical reasoning, however according to surveys done by Rencic et al. in 2017 [5] on clerkship directors in United States medical schools, and Kononowicz et al. in 2020 [6] on international medical education community, reported that most of the respondents did not have explicit longitudinal clinical reasoning curriculum in their centers. Respondents also suggested that clinical reasoning should be introduced early in medical school and not only taught during the clinical years. Among the barriers identified were the lack of faculty expertise to teach clinical reasoning concepts, lack of awareness on the need to have explicit clinical reasoning teaching and the perceived notion that clinical reasoning could not be taught. Faculty development with train-the-trainer program in clinical reasoning was agreed on by majority of the respondents [6] as one of the possible means to overcome the barriers. A qualitative study done by Angus et al. in 2018 [7] in experienced clinical educators shared similar notion, in which clinical reasoning was difficult to teach and might be impossible to teach in some students. This was attributed to the lack of awareness on own clinical reasoning process in experienced educators [7–8].

The teaching of clinical reasoning is advocated to begin early in medical schools, which is from the pre-clinical years [5,8] for students to be able to appreciate and apply basic science knowledge learnt. Pre-clinical and clinical teaching and learning differ as students have a more scheduled and structured teaching during pre-clinical as compared to a more active and independent learning during clinical years [9]. To effectively teach clinical reasoning during pre-clinical years, educators involved need to comprehend and be well-versed in the art of clinical reasoning teaching. Some educators in the pre-clinical years may not be trained in medicine as their first degree leading to different views and perspectives compared to their clinical counterparts. Clinical educators and non-clinical medical education experts have reported a difference in their rank of the educational principles for successful clinical teaching based on a Delphi study [10]. The mismatch between clinical and non-clinical educators may have an impact on the design and development of any faculty development program.

To the best of our knowledge, there are a limited number of qualitative studies on educators’ perception on clinical reasoning, and these have primarily focused on clinical educators in the West. It will be very interesting to explore the perceptions of both pre-clinical and clinical educators on clinical reasoning, especially in an Asian context as culture plays a part in shaping motivation and attitudes that impacts learning [11,12]. It should be noted that Asian students were more dependent on their educators when compared to Western students who were more independent in their learning [13,14]. Strict educators in Asian education environment were shown to have positive association with students’ motivation whereas it showed negative association in Western students, and this may be due to the Asian culture of obedience and respecting elders and those with authority [15]. The culture of uncertainty avoidance in Asian students and the belief that any knowledge should be memorized to be understood might have a negative outcome on the development of critical thinking and clinical reasoning skills [13]. However, most medical institutions in Asia adopts the Western curriculum with minimal cultural adaptation. This study seeks to answer this question: What are the perceptions and understanding of pre-clinical and clinical educators of a Malaysian public medical institution on clinical reasoning? This study aims to explore the pre-clinical and clinical educators’ perception on clinical reasoning, whether there is any difference in the perception between pre-clinical and clinical educators, and between Asian and Western educators.

## Methodology

To obtain educators’ perception on clinical reasoning, a semi-structured interview was conducted with chosen pre-clinical and clinical educators from Faculty of Medicine, Universiti Teknologi MARA (UiTM), Malaysia via purposive sampling method. The educators’ list was obtained from the Human Resource Department following which a discussion was done among the researchers to identify suitable educators. The sampling frame included pre-clinical and clinical educators from various disciplines. For pre-clinical, the educators were either medically trained or non-medically trained, teaching basic science subjects or laboratory-based subjects. For clinical educators, the participants were from medical and surgical-based specializations. The selected participants must fulfil the following criteria: 1) more than 5 years teaching experience, and 2) active involvement in teaching and assessment of undergraduate medical students. For pre-clinical educators, teaching involves lecture, problem-based learning (PBL), practical, and tutorial, whereas teaching in clinical years involves bed side teaching, ward rounds, clinic attachment, and multi-disciplinary seminars.

Invitations were sent to the prospective participants via official institutional email or personal telephone conversation. Interview date, time and place were scheduled based on the participants’ request. Before the start of each interview session, the author briefed the participants on the details of the Participant Information Sheet (PIS) which included the working definition of clinical reasoning used in this study [1,3], objectives and benefits of the study, as well as the interview process. Written informed consent was then obtained, and participants were assured of their confidentiality. The interview questions consisted of participants’ academic and work background, teaching experience in clinical reasoning, and educational strategies to improve clinical reasoning. The participants were encouraged to share their honest perception to each question posed. The questions were open-ended and adapted from Angus et al., 2018 [7] and Addy et al., 2016 [16]. A comprehensive interview protocol, including the full interview questions and prompts was developed and discussed earlier among the authors. A pilot interview was done with one of the faculty members to see the feasibility and appropriateness of the interview protocol. The interview questions are shown in Table 1.

**Table 1:** Interview questions and prompts (adapted from Addy et al., 2016 and Angus et al., 2018).

**Interview Questions** Please tell me your academic background and your current work background What is your role or involvement in the teaching of undergraduate medical students? Can you tell me what do you understand with the term ‘clinical reasoning’? What is your approach to clinical reasoning? What is your style of reasoning? Have you been able to apply clinical reasoning in your teaching? In your course of applying clinical reasoning in your teaching, do you face any difficulty? What do you think of our medical students clinical reasoning skills? Do you think that clinical reasoning is a skill that can be taught? In your opinion, how can we improve clinical reasoning skills in medical students? Do you think it is important for teachers to know how to teach clinical reasoning to students? Is there a need to have a training for teachers? What would you like to see in that training? What would you like to be taught on? What would you like to learn?

The interviews were conducted from 5^th^ July 2022 to 5^th^ September 2022 and the entire interview session was digitally audio recorded. Interviews were conducted until a saturation of data was achieved. The average time for the interview session was 26 minutes. Subsequently, the interviews were manually transcribed, and a copy of the transcript was sent to each participant via email to ensure accuracy.

Thematic analysis was chosen as the interview data were inductive in nature, the themes were at semantic level and fell into the essentialist or realist epistemology. The thematic analysis followed a step-by-step process as follows; familiarization with the data, generating initial codes, searching for themes, reviewing the themes, defining and naming themes, and producing the report [17]. The coding was done using Atlas.ti by the first and third author, the themes were identified and checked within and across the interviews of each participant. All the authors reached a consensus on the themes, and exemplar quotes for each theme were extracted.

This study was approved by Universiti Kebangsaan Malaysia (UKM) Research Ethics Committee (UKM/PPI/111/8/JEP-2022-219) and written approval was obtained on 15 April 2022.

Reflexivity statement: All team members came from a diverse background in academics. The first author was a pre-clinical educator where the study was conducted and was an early career researcher and pursuing a doctoral degree in the field of medical education. The first author was responsible for interviewing all the participants who were her colleague. The relationship between the first author and the participants was purely professional. The second and third authors were an otorhinolaryngologist and a medical educator respectively and had considerable amount of experience conducting research in the field of medical education.

## Results

### Interview participants

Fifteen educators from diverse background in the Faculty of Medicine, UiTM Malaysia was interviewed for this study. Seven participants were involved in teaching the pre-clinical years, with five participants from the basic science subjects and two participants from laboratory-based subjects. In terms of their first degree, four participants held medical degree while three had other qualifications. Eight participants were clinical educators with five from medical-based discipline, and one participant each from surgical-based, community-based and emergency medicine discipline.

The participants exhibited a diverse range of knowledge on clinical reasoning. Some had never even heard the term ‘clinical reasoning’, some had minimal understanding, and some had read about clinical reasoning before.

### Themes

Eight (8) key themes were identified on perception of pre-clinical and clinical educators on clinical reasoning, with the subthemes were shown in italics. Knowledge was recognized as an important component in clinical reasoning, along with experience. Educators also agreed that teaching clinical reasoning was feasible but difficult, thus the necessity for training in clinical reasoning. Additionally, themes such as early introduction of clinical reasoning, its incorporation in various teaching and learning modalities and attributes of the educators were also identified in this study. The identified themes and the excerpts of each theme are as follows:

#### 1. Knowledge is important.

Both pre-clinical and clinical educators agreed that knowledge in basic science subjects and clinical medicine subjects was one of the *important aspects* of clinical reasoning. In addition of having the right and relevant knowledge, the *application* of basic science knowledge and *integration* of knowledge from various disciplines were key to good clinical reasoning.

*“Clinical reasoning is easier… [in students] whose knowledge are already there. … able to link, to reason it with whatever information that they have.”.* (Participant 13)

*“ they appreciate that [clinical reasoning] … they are able to … link the basic sciences to the current clinical topics.”* (Participant 16)

Medical students were not the only ones who needed to have the depth and breadth of knowledge as participants agreed that *educators too need to be equipped* with extensive basic and clinical medicine knowledge, and *knowledge on clinical reasoning itself*.

*“I always feel like I have to improve myself … we ourselves as lecturers need to understand how to teach students … can trigger their thinking”* (Participant 7)

*“I think the teachers need to be well equipped first with clinical reasoning … to correlate with what we are teaching and to whom … I think the teachers need to be well versed first.”* (Participant 2)

#### 2. Experience is essential.

Apart from knowledge, participants perceived experience as another important factor in the development of clinical reasoning. Educators agreed that they have better knowledge and clinical reasoning skills when compared to medical students, which was attributable to the years of experience that they have.

*“I don’t have difficulty in applying clinical reasoning … because I’m very familiar to it … to train the medical students … I would have to come down to their level … rather than they come up to my level.”* (Participant 9)

*“When I teach the undergraduates … I understand they are very young, very novice and they couldn’t correlate.”* (Participant 11)

#### 3. Teaching clinical reasoning is feasible.

Pre-clinical and clinical educators perceived that clinical reasoning was a skill that can be taught to the medical students, albeit with its own challenges and difficulties.

*“Lots of things we can learn … it’s only up to us whether we want to learn or not … everything that is related to skill … can be taught.”* (Participant 5)

*“Yes, it’s a skill … it [clinical reasoning] definitely can be taught.”* (Participant 11)

As any other skills, clinical reasoning was a skill that also needed to be *practiced, mentored, and guided* by the educators.

*“It [clinical reasoning] need to be coached … need to have guidance … to make them [students] understand and relate more.”* (Participant 4)

*“Sometimes we assumed that clinical reasoning can be learnt on their own, but they cannot be learnt on their own, they got to be guided.”* (Participant 9)

#### 4. Teaching clinical reasoning is difficult.

Although it was feasible to teach clinical reasoning, it had its own difficulty. The perceived difficulty in teaching clinical reasoning to medical students were coming from the *educators, faculty/institution* and even the *students* themselves. Some educators were not sure with their own understanding of clinical reasoning.

*“I am not sure whether my definition of clinical reasoning is correct or not.”* (Participant 2)

*“It can be done if … everybody is on the same page … everybody must say the same thing, do the same thing.”* (Participant 6)

*“It will be difficult … because it’s not easy to standardize clinical reasoning … different specialist has different style.”* (Participant 10)

The *amount of work* and *multiple roles* in academic, administrative, and clinical settings led educators to *time constraints*.

*“The teaching sessions and … we are also involved in developing questions, vetting, examiners … and also supervising their elective projects.”* (Participant 13)

*“I was the clinical coordinator, I also involved in assessment in the professional exam … end of posting exam. I teach the Year 3 and Year 5 … seminars, bedside teachings, ward rounds and also clinical sessions … in the outpatient clinic.”* (Participant 11)

*“I think the biggest problem is time… I would do my bedside teaching in hospital … up to 11 o’clock … I would take the students from 11.30 up to 1 o’clock. But sometimes … there are emergencies and I have to leave them [students]”* (Participant 11)

Another challenge in teaching clinical reasoning came from the faculty/institution in which *students’ timetable, insufficient manpower* and the *logistics* of having branches in different sites were cited by the participants.

*“It’s too much to take in... they [students] haven’t got time to digest because … they are jumping from one rotation to the other … perhaps they need that time where they don’t receive any new things and reflect on what they did.”* (Participant 15)

*“Our students … are based in Sungai Buloh and Selayang. And then the hospital is in Puncak Alam … the travelling … is very time consuming. … they will miss out a lot on patient exposure”* (Participant 4)

*“The manpower is not enough … you have to do all … you have to teach … and also the clinical work that we have.”* (Participant 11)

In terms of students, participants perceived that critical thinking skills in students were less than desirable and they were *unable to see the bigger picture* (Participant 2). However, students’ interest and eagerness in learning were the positive attributes that could aid in learning clinical reasoning.

*“Our students are not really trained in thinking as much and also thinking outside the box.”* (Participant 12)

*“Trying to regurgitate what they have heard in the lectures … they are not really thinking in a stepwise clinical reasoning.”* (Participant 8)

*“When they don’t prepare it will be very difficult … maybe they have the drive and determination.”* (Participant 6)

*“It’s [clinical reasoning] still poor. But … their interest is very great … because it is something that they want to know.”* (Participant 10)

#### 5. Educators need training in clinical reasoning.

Due to the uncertainty in understanding clinical reasoning coupled with the difficulty stemmed from the educators, faculty and students as perceived by the participants; a training in clinical reasoning was very much needed.

*“The art of teaching must be taught … we assume when you become a lecturer, you know how to teach. But it’s not the same. You don’t have any formal training.”* (Participant 9)

*“The way that we empower our educator is actually to train them … we do have training but it’s not adequate.”* (Participant 11)

Participants believed that to be able to teach clinical reasoning to students, the educators need to understand and be well-versed first with the concept of clinical reasoning.

*“If we don’t know how to teach … or how to guide … how can the students know if we don’t know. We must know first.”* (Participant 6)

*“The teachers must be able to do clinical reasoning themselves and they must be able to know how to show the students how to do it.”* (Participant 9)

#### 6. Early introduction to clinical reasoning.

Pre-clinical and clinical educators perceived that students should be introduced and taught about clinical reasoning quite early in the medical school, even during *pre-clinical years*.

*“Many lecturers felt that clinical reasoning only important during clinical years which I guess it is totally wrong. It should start as early as possible.”* (Participant 5)

*“Can be taught … as early as Year 1 … even though they are not seeing patients in Year 1, but they are learning the process”* (Participant 9)

Following the early introduction in the pre-clinical years, the teaching of clinical reasoning should have the continuity until the clinical years, making the gap between pre-clinical and clinical year smaller and more manageable.

*“There is … [difficulty] with the pre-clinical … quite stressed in the clinical years because you have to have a good basic to apply that in clinical.”* (Participant 6)

*“As pre-clinical lecturer, I also don’t know what is the expectation from clinical lecturers … important … that what we teach during pre-clinical years … they can apply in clinical years.”* (Participant 7)

#### 7. Incorporating clinical reasoning in various teaching and learning methods.

Clinical reasoning should not only be taught during bedside teaching. It could be incorporated in other teaching and learning methods too during the pre-clinical and clinical years.

*“Other sessions that we can do like case-based learning or team-based learning which can improve their clinical reasoning.”* (Participant 3)

*“Can incorporate into various teaching delivery method … in tutorial … during DSL, during practical … we can incorporate clinical reasoning in these.”* (Participant 5)

Didactic teaching of clinical reasoning which was the main method used in pre-clinical years was perceived poorly by the participants. The teaching of clinical reasoning should be conducted in an *active environment* with real scenario or cases.

*“Give them problem-based learning, case-based discussion moderated by them and facilitated by us … more of interactive discussion … more of students participation.”* (Participant 9)

*“Work in groups … come up with a team-based learning … try to solve a problem and looking back at the pre-clinical basic sciences and try to link it with the current clinical problems.”* (Participant 16)

The use of *technological advances* to assist in teaching clinical reasoning was the sentiment perceived only by the clinical educators, and not by the pre-clinical educators involved in this interview.

*“They can practice by themselves … study at their own convenience … simulated or … virtual patient … I think we can do that also.”* (Participant 4)

*“I think we can actually put things together from pre-clinical to clinical is to have a simulation based medical education … we allow the students to … do mistake in a very safe environment.”* (Participant 16)

Participants perceived that students need to be *informed explicitly* on the teaching of clinical reasoning. Educators also need to share the exact learning outcome expected of the students and clear instructions must be made.

*“You need to … introduce what you mean by clinical reasoning … tell them what you are going to do in the clinical setting … what they [students] need to do.”* (Participant 10)

*“I let them know my ground rules … we must be clear … our instructions to students must be clear.”* (Participant 5)

#### 8. Attributes of educators.

The participants perceived that educators need to be *receptive of changes* and have the drive to always improve themselves for the better.

*“People [educators] … must be receptive to what we want to teach.”* (Participant 6)

*“I feel like I need to improve myself … have an idea … to teach the students because students learn differently.”* (Participant 7)

The educators need to guide the students on *knowledge integration*, *spur the critical thinking* of students and to always *explain their actions* and train of thoughts.

*“Even though the discussion mainly focused on the pharmacological part, but we would touch upon the physiological or pathological part of the disease so they could understand.”* (Participant 3)

*“I try to make them think … ‘why is this information important in terms of getting a diagnosis’ and so on.”* (Participant 13)

*“Guiding them on the correct way to think … thinking skill … it is difficult.”* (Participant 6)

*“It [clinical reasoning] will improve when you … explain, instead of … directive.”* (Participant 10)

Besides the obligation of the educators in the cognitive aspect of learning, educators should also be equipped with related *soft skills*. Participants highlighted the need of educators to be well versed in asking and probing questions, facilitation skills, giving meaningful feedback to students and reflection.

*“You have to ask the students the correct questions … it will trigger their brain. ‘Why does this happen’ … ‘How does this happen’ ….”* (Participant 9)

*“We give feedback … ‘you did well here, but you have some components that you want to improve on, and therefore in the next session we will see whether you have improved in this part’.”* (Participant 16)

*“I think … with a lot of reflection on why we do things or why we do what we do or why this was decided in that way.”* (Participant 12)

*“Must know how to give feedback, how to reflect on their practices … how to motivate, how to become facilitator, how to coach … how they [educators] can go towards their professional identity formation.”* (Participant 9)

## Discussion

Referring to the identified themes from the interviews, the perception of clinical reasoning by pre-clinical and clinical educators were almost similar. Even though the educators had diverse academic and working background with some had no experience in clinical work, they emphasized on two important factors: knowledge as the foundation of good clinical reasoning and the role of experience in the evolution of clinical reasoning in an individual. To the best of our knowledge, the literature on pre-clinical and clinical educators’ perception on clinical reasoning in an Asian health education landscape was lacking and these findings showed that educators in Faculty of Medicine, UiTM Malaysia were in agreement with most of the international health educators in terms of important factors in clinical reasoning.

Knowledge was perceived by the participants as the utmost important aspect in clinical reasoning, encompassing basic science, clinical medicine and even knowledge on clinical reasoning itself. According to the participants, the importance of knowledge was not only for the development of clinical reasoning in students, but also in educators. This view on the importance of knowledge aligned widely with clinical reasoning literatures. Both content knowledge and cognitive processes used in problem solving were identified as key components in clinical reasoning [1] and inadequate knowledge could lead to diagnostic error [18]. More crucial than the depth of knowledge was the organization of knowledge for the ease of recalling key concepts during problem-solving [18]. However, a study done by Kiesewetter et al. in 2016 [19] found that knowledge alone was not the only factor in solving clinical cases, instead, it was the goal-directed application of knowledge. They concluded that conceptual and strategic knowledge decreased with an increase in metacognitive knowledge along the course of solving clinical cases. Furthermore, knowledge reorganization was shown to have small but consistent benefits in reducing diagnostic errors when compared to recognition of biases in students [20].

Experience perceived as another important aspect by the participants, as clinical reasoning improved with exposure and time. Despite pre-clinical educators not having patient encounters, they recognized the importance of experience in developing clinical reasoning. This may be credited to the exposure to various clinical cases that they encountered during the preparation of teaching and assessment sessions that made them more familiar with the concept of clinical reasoning. This perception showed that the educators were aware of their own clinical reasoning development unlike the findings by Angus et al. in 2018[7], where experienced educators might have lacked the awareness of their own clinical reasoning development. The importance of experience also aligned with the concept of dual process theory (DPT) which posits two types of reasoning systems: System 1 and System 2 [21]. System 1 was an intuitive and unconscious approach or also known as non-analytical whereas System 2 was more analytical, conscious, and deliberate [21,22]. System 1 approach was used mostly by experts as experience over time would make the pattern recognition of similar diseases easier [21]. The role of experience could be used to inculcate clinical reasoning in students, as exposure to simple and complex clinical problems during teaching sessions would provide them with the essential experiences needed [23]. This exposure would direct the students to develop their own illness scripts of different diseases and together with deliberate practice in comparing and contrasting diseases, clinical reasoning could be strengthened [24].

Clinical reasoning itself was viewed by participants as a skill, thus it was something that can be taught. Clinical reasoning was classified under procedural knowledge, similar as blood pressure measurement and other clinical skills. The development of procedural knowledge involved the crucial steps of coaching, constructive feedback, and deliberate practice [1]. All the participants in this study held a positive view that clinical reasoning could be taught, but with its own difficulties. This view was shared by Angus et al. in 2018[7], in which the participants of a qualitative study admitted that clinical reasoning was not easy to teach. The perception that clinical reasoning cannot be taught was seen in some participants of surveys done by Rencic et al. in 2017 [5] and Kononowicz et al. in 2020[6].

Previously, three forms of barriers and challenges in teaching clinical reasoning were identified which were content, environment and expert or teacher factors [8]. In this current study, three form of difficulties in teaching clinical reasoning were identified: educator, institution, and student factor. Participants viewed the high workload, lack of personal understanding, and disconcerted goals or mismatched expectations on clinical reasoning among educators as the difficulties that stemmed from the educators’ side. To be able to teach clinical reasoning meaningfully, educators need to have the same understanding on definition and concepts of clinical reasoning [25] and a common goal. It was shown that clinical teachers had many roles, worked in a demanding and complex environment, and they were not given the proper orientation or preparation before assuming their tasks [26]. Pre-clinical educators also had a myriad of tasks unrelated to teaching and research such as administrative tasks which took a toll on them and could negatively impact their abilities to fulfil assigned tasks. There’s a need for the educators to have the same understanding, goal, and outlook on the teaching of clinical reasoning and this could be achieved by uniformed training.

Students’ timetable, insufficient manpower and multiple campuses were perceived as the difficulties that arose from the institution’s side. The medical curriculum in the place of this study consisted of many core subjects and there were also some additional subjects deemed necessary by the university for the students to learn in their pre-clinical years, further reducing the time for self-directed learning. In many Asian educational systems, significant emphasis was placed on assessments and rote memorization, which often leads to student timetables being filled with lectures and classes. Some of the senior educators had resigned from the public service and worked in the private sector due to a variety of factors; be it for financial gains or self-fulfilment, thus creating a void in the public medical institution. Higher number of educators who were experts in their respective fields need to be recruited to reduce the burden and prevent burnout amongst educators. To accommodate the large number of students and making sure each student had maximum clinical exposure, Faculty of Medicine UiTM had multiple campuses targeting different health education aspects such as urban and rural health. Even though this arrangement did help with students’ educational needs, it posed as a difficulty in terms of logistics to students and educators.

Gradual change in the curriculum, such as implementing curriculum structured on clinical representation rather than system-based [1], or even spiral curriculum [22] could be beneficial to address the issue of student timetable. Lack of curricular time was cited as a significant barrier in implementing clinical reasoning curriculum [5,6] and institution could integrate the teaching and learning clinical reasoning to be more explicit in existing curriculum along the years [27]. The institution’s role was beyond curriculum, as policies and matters related to the well-being of educators was also under its purview, and this included the appointment of more qualified manpower. The issue of educators resigning from public medical institutions and the logistics implication from multiple campuses were beyond the scope of the institution and needed reform from higher governance. However, the current study did highlight these difficulties and it was hoped that it brought awareness for the institution to engage the relevant parties for further discussion.

Students were perceived to have less than desirable critical thinking skills that hindered them from knowledge application. Participants perceived students with high critical thinking skill, asking the right questions and always explaining their train of thoughts as having the desirable qualities to instil clinical reasoning. Achieving this may require a change in the curriculum and teaching and learning modalities towards more active learning approach.

As teaching clinical reasoning proved to be a challenge in pre-clinical and clinical years, participants believed that educators would benefit from training in clinical reasoning. They believed that educators need to really understand and be proficient in clinical reasoning themselves before imparting it to students. Unfortunately, train-the-trainer programs in clinical reasoning were not readily available in most medical institution, and these programmes were deemed necessary by most of the respondents surveyed by Kononowicz et al. in 2020 [6]. Existing train-the-trainer programs were mostly targeted for clinical educators [28,29] and not tailored for pre-clinical educators. Feedback from these programs were mostly positive, with participants perceiving an increase in their confidence level and committing to changes in teaching clinical reasoning [29]. A train-the-trainer program curated and developed for both pre-clinical and clinical educators might be the solution.

In teaching clinical reasoning, participants agreed that it should be taught as early as possible; even during the first year of the pre-clinical phase, incorporated in various teaching and learning modalities with educators having a significant role. This viewpoint was also shared by the participants in the survey done by Rencic et al. in 2017 [5], in which the respondents viewed that clinical reasoning should be taught in all phases of medical education. Early introduction to clinical reasoning might make the pre-clinical students appreciate the importance of basic science subjects [8,30]. However, not all aspects of clinical reasoning should be taught in the early years, as the content and approach need to be tailored to the maturity and knowledge level of the students [24,25]. In view of early introduction to clinical reasoning, pre-clinical educators should be equipped with the appropriate knowledge on clinical reasoning, and this made the need to have a train-the-trainer program much more important.

To enhance the teaching of clinical reasoning, it should be incorporated into various teaching and learning methods, preferably in an active learning setting. Bedside teaching, problem-based learning and virtual patients were some of the methods in which clinical reasoning should be taught [6]. Blended learning strategies encompassing active and collaborative learning principles such as case-based discussion and flipped classroom were useful for students to discuss, apply and reflect on their learning [30]. Participants in this study were not particularly agreeable with lectures or didactic teaching as an effective mode to teach clinical reasoning and this view was also shared in a survey by Kononowicz et al. in 2020 [6].

Interestingly, clinical educators in the current study pointed out the role that technological advances could play such as gamification and simulation in the teaching of clinical reasoning. This view however was not shared by the pre-clinical educators. This difference in view could be due to the way pre-clinical years were taught; student timetable had mostly lectures and less opportunity to incorporate simulation. Pre-clinical educators in this study institution needed to deliver the learning objectives identified for each topic in a specified time frame, giving rise to compressed lecture laden with information. This echoed the findings by Findyartini et al. in 2016 [13] in which Asian academics emphasized on the importance of content expertise and the process of knowledge transfer.

Whereas in clinical years, the use of simulation in clinical skills lab was quite substantial to provide the students with a safe environment to solve clinical cases. Students have been utilizing clinical skill laboratory to practice procedural knowledge such as blood pressure measurement and insertion of nasogastric tubes under the supervision of clinical educators. The nature of procedural knowledge that was predominant in clinical years also played a part in clinical educators’ agreement on the benefits of technological advances. The use of virtual patient was shown to improve clinical reasoning skills in students as it could complement case-based discussion, more so if the number of cases in the hospital were limited [31]. It also provided students with a safe environment in which making a diagnostic or management mistake was not harmful, thus prompting clinical educators to utilize these advances. The pre-clinical educators need protected time to allow for more creativity in incorporating technological advances to inculcate clinical reasoning in their teaching.

Participants in this study also agreed that being an educator was a significant responsibility and should be honoured by giving their utmost dedication in teaching. They were responsible for the cognitive aspect or imparting knowledge to students. Apart from that, they needed to be equipped with the soft skills to enhance teaching. Educators needed to be a coach; to be a role model, to motivate and to give feedback, and these attributes might be one of the important roles of an educator [32]. A review done by Sutkin et al. in 2008 [33] found that non-cognitive attributes were perceived as characteristics of good clinical teachers compared to cognitive attributes. This showed the vast role that educators undertook as a mentor, role model, facilitator, and others.

This study had its own limitation in which the study was conducted in a single centre and the findings obtained were specific to this medical faculty, and it may not be suitable to be generalized to other medical institutions. It is of interest to see the perception of pre-clinical and clinical educators of other public and private medical institution in Malaysia on clinical reasoning and to compare it with the perception of educators in other parts of Asia and elsewhere. To really retest and refine the findings in this study, perhaps a quantitative study on faculty development needs on the rest of the educators would be ideal and should be done in the near future.

## Conclusion

Pre-clinical and clinical educators had somewhat similar perception and understanding on clinical reasoning, the only difference was on the perceived role of technological advances in aiding the teaching and learning of clinical reasoning. As they perceived that clinical reasoning can be taught albeit its difficulties, a train-the-trainer programme tailored for pre-clinical and clinical educators could be of assistance in inculcating clinical reasoning in various teaching and learning modalities. Promoting the teaching of clinical reasoning was the concerted efforts by the institution, educators, and students; and the importance of understanding clinical reasoning by these stakeholders were undeniable.

## Supporting information

S1 File COREQ Checklist. (PDF)

S1 Table Codes and themes. Codes and themes from interview sessions.
(DOCX)
