# Supplementary material for: Exploring the perception of pre-clinical and clinical educators on clinical reasoning: A qualitative study
Source: PLoS One. 2025 Mar 21;20(3):e0320220. doi: 10.1371/journal.pone.0320220 (PMC11927892; doi:10.1371/journal.pone.0320220)
Supplement: S1 Table — Codes and themes from interview sessions. (DOCX) [file pone.0320220.s002.docx]

Codes and themes from interview sessions

| Codes | Minor themes | Major themes |
| --- | --- | --- |
| - importance of basic science knowledge- importance of CR process- importance of knowledge- integration of knowledge- must have basic science and clinical knowledge- must have continuity from pre-clinical to clinical- need basic knowledge- teacher must have knowledge- understand basic science knowledge first | Importance of knowledge is undeniable | Knowledge is important |
| - application of basic knowledge- importance of seeking verified information- no integration of knowledge in clinical students- no integration of knowledge in pre-clinical students- students can't remember basic knowledge | Integration and application of knowledge |  |
| - CR differs in students and teachers- CR in students varies- CR is better with experience- CR is less in pre-clinical students- CR trains competence- early clinical students are more reserved- importance of experience- mentoring system- students have less CR experience- teachers need to understand students are novice- learning through experience | Experience is essential | Experience is essential |
| - CR can be taught- CR is an effort by both teachers and students- do not blame students- effort from student is important- faculty plays a big role- students eager to learn | Teaching CR needs effort from everyone | Teaching CR is feasible |
| - no follow-up on difficult learners - teachers have different styles of teaching- too much to learn and teach- unable to achieve LO- unsure of own CR definition - unsure of own CR teaching | Teaching CR is feasible, but difficult |  |
| - academic burden- administrative tasks- expectation of clinical teachers- experts have more responsibilities- teachers fatigue- teachers have multiple roles - teachers wellbeing is important  - time is a constraint - unsure of own teaching technique- unsure of own CR definition - unsure of own CR teaching | Educators are burdened | Teaching CR is difficult |
| - multiple campuses is a barrier- packed timetable- more manpower needed - time is a constraint | Institutional difficulties |  |
| - students are spoon fed- students are unable to apply knowledge- students are unable to integrate knowledge- students are unable to relate- students are unable to see the bigger picture- students need to have higher thinking skills- students need to think critically | Difficulties that stem from students |  |
| - CR needs to be guided - new teachers lack experience - no proper training- teachers must be trained- teachers must know how to teach- teachers need guidance- teachers need to improve themselves- teaching must be standardized- everyone must have the same goal | Training is needed and maybe beneficial | Educators need training in CR |
| - teachers need to guide students on knowledge integration- teachers need to know CR first- teachers need to know how to incorporate CR | Educators need to know how to teach CR |  |
| - CR in various T&L- early exposure to CR- learning through practical- less opportunity to learn and apply CR | Students should have early exposure to CR | Early introduction to CR |
| - CR in various T&L- early exposure to CR- learning through practical | CR in various T&L with early exposure | Incorporating clinical reasoning in various teaching and learning methods |
| - active learning- CR is better taught in active learning- CR needs practice- hands-on learning- importance of PBL- learning through discussion is better- passive teaching is not encouraged- scenario-based teaching- students like hands-on session- way of teaching not really changed- students learn better in small groups | Active learning with real case |  |
| - importance of virtual patients- incorporate technology in teaching- simulation teaching | Incorporating technology/simulation in teaching CR (only in clinical educators) |  |
| - asking relevant questions- feedback to students is important- probing questions- reflection is important- teachers must explain- teachers must have soft skills- teachers must know how to facilitate- teachers must know how to motivate- teachers must reflect- teaching must spur critical thinking | Educators play a big part | Attributes of educators |
